# Elevated Levels of Circulating lncRNAs LIPCAR and MALAT1 Predict an Unfavorable Outcome in Acute Coronary Syndrome Patients

**DOI:** 10.3390/ijms241512076

**Published:** 2023-07-28

**Authors:** Teodora Barbalata, Loredan S. Niculescu, Camelia S. Stancu, Florence Pinet, Anca V. Sima

**Affiliations:** 1 Lipidomics Department, Institute of Cellular Biology and Pathology “Nicolae Simionescu” of the Romanian Academy, 8 Bogdan Petriceicu Hasdeu Street, 050568 Bucharest, Romania; teodora.barbalata@icbp.ro (T.B.); loredan.niculescu@icbp.ro (L.S.N.); camelia.stancu@icbp.ro (C.S.S.); 2 U1167-RID-AGE-Facteurs de Risque et Déterminants Moléculaires des Maladies Liées au Vieillissement, Institut Pasteur de Lille, Université de Lille, INSERM, CHU Lille, F-59000 Lille, France; florence.pinet@pasteur-lille.fr

**Keywords:** long non-coding RNA, LIPCAR, MALAT1, miR-155, miR-142, coronary syndrome, stable angina, unstable angina, STEMI, biomarker

## Abstract

Coronary artery disease (CAD) is a leading cause of mortality worldwide. In this study, we aimed to assess the potential of plasma long non-coding RNAs (lncRNAs) LIPCAR and MALAT1 and microRNAs (miRNAs) miR-142-3p and miR-155-5p to discriminate unstable CAD patients from stable ones. 23 stable angina (SA), 21 unstable angina (UA), and 50 ST-segment elevation myocardial infarction (STEMI) patients were enrolled; their plasma was collected. ncRNA plasma levels were evaluated using RT-qPCR. All measured ncRNA levels were significantly increased in UA patients’ plasma compared to SA patients’ plasma and in STEMI-with major adverse cardiovascular event (MACE) patients’ plasma vs. STEMI-without MACE patients’ plasma. ROC analysis showed that increased levels of LIPCAR and MALAT1 were associated with UA, and the prognostic model improved with the addition of miR-155-5p levels. The assessed lncRNAs discriminated between hyperglycemic (HG) and normoglycemic (NG) UA patients, and they were associated with MACE incidence in STEMI patients; this prediction was improved by the addition of miR-142-3p levels to the ROC multivariate model. We propose LIPCAR and MALAT1 as effective diagnostic markers for vulnerable CAD, their association with HG in UA patients, and as robust predictors for unfavorable evolution of STEMI patients.

## 1. Introduction

Cardiovascular diseases (CVDs) are the leading cause of death worldwide, despite tireless efforts to find better diagnostic and prognostic tools. Understanding their underlying molecular mechanisms is key to the treatment of patients. Some of the most threatening CVDs are coronary artery diseases (CADs), which can be classified based on their severity as stable angina (SA), unstable angina (UA), and myocardial infarction (MI), the latter two being collectively known as acute coronary syndrome (ACS) [1]. Although there are some parameters currently used in the clinic to evaluate a patient’s risk to develop MI, they have proven to be insufficient to discriminate CAD patients at risk [2]. There is an inherent need to find new and accurate prognostic biomarkers to predict CAD patients’ evolution.

One of the emerging classes of promising molecules is long non-coding RNAs (lncRNAs), which are non-coding RNAs (ncRNAs) usually up to 200 nucleotides long. They were first observed in humans during cancer research [3], but they were later found to be implicated in CVD development as well [4]. Another class of ncRNAs that shows great potential as diagnostic and prognostic biomarkers for CVD is microRNAs (miRNAs) [5], which are short ncRNA sequences usually 20–23 nucleotides long. Considerably more widely studied than lncRNAs, many miRNAs have been described in the literature as being involved in the regulation of many pathways relevant to CVD, such as those involved in cardiomyocyte contractility, hypertrophy, or fibrosis [6,7].

The regulation mechanisms of lncRNAs are fairly diverse and less studied. They are known to act by either direct interaction with chromatin, thus affecting its condensation state, or by binding to histones or transcription factors [8]. In the cytoplasm, lncRNAs have multiple mechanisms of action, such as inhibition of gene expression by binding to RNA-binding proteins specific for mRNA or even to mRNA molecules directly [9]. Alternatively, it has been reported that they can bind to and block miRNAs, forming so-called “miRNA-lncRNA sponges” that diminish the regulatory effects of miRNAs [10]. Another difference between lncRNAs and miRNAs is that the former are significantly less conserved than the latter, their expression being more tissue-specific and, therefore, they are less abundant in circulation [11]. 

Two lncRNAs that have been demonstrated to regulate different pathways relevant to CVD are uc022bqs.1 (long intergenic noncoding RNA predicting cardiac remodeling and survival of patients with heart failure—LIPCAR) and metastasis-associated lung adenocarcinoma transcript 1 (MALAT1). LIPCAR is a relatively newly discovered mitochondrial lncRNA [12] that has been described to have a role in heart failure [13,14]. Although little is known about the pathways in which it is involved, there are some in vitro data suggesting that LIPCAR is implicated in endothelial cell and smooth muscle cell proliferation and migration [15,16]. MALAT1 is one of the most studied lncRNAs [17]. Although it was originally observed in cancer, it has been described in relation to CVD as well [18]. For example, it has been linked to lipid accumulation in macrophages treated with oxidized LDL (oxLDL) [19], suggesting a potential role in atherosclerotic plaque formation. MALAT1 was also found to be implicated in endothelial cell dysfunction, as demonstrated in HUVECs exposed to oxLDL [20].

Two microRNAs that are known to be involved in the regulation of CVD are mir-155-5p and miR-142-3p. miR-155-5p has been shown to promote atherosclerosis by affecting endothelial function [21], while elevated levels of miR-142-3p have been linked to major adverse cardiovascular events (MACEs) in ST-segment elevation myocardial infarction (STEMI) patients [22]. Both miR-155-5p and miR-142-3p have been reported to be regulated by MALAT1 in vitro via the “sponging” effect [23,24].

In the present study, we analyzed the distribution of LIPCAR, MALAT1, miR-155-5p, and miR-142-3p in the plasma of SA, UA, and STEMI patients in order to investigate their potential to discriminate ACS patients at risk. For SA and UA patients, the risk was considered to be hyperglycemia (HG), while for STEMI patients, MACE was considered to be the risk. We therefore correlated their circulating levels with HG and MACE, respectively.

## 2. Results

### 2.1. Plasma Biochemical Parameters of SA, UA, and STEMI Patients

Plasma lipid and oxidative stress parameters of SA, UA, and STEMI patients, with or without MACE, are presented in Table 1. Our results showed that patients with vulnerable CAD (UA and STEMI patients with MACE, respectively) had an aggravated biochemical plasma profile compared to SA and STEMI patients without MACE. 

We found statistically significant increased levels of total cholesterol (TC) (by 21%, *p* = 0.011), apolipoprotein E (ApoE) (by 33%, *p* = 0.024), and C-reactive protein (CRP) (2-fold, *p* = 0.015) in UA patients versus SA patients (Table 1). Of note, the observed CRP levels in ACS patients were elevated compared to the standard values. We further observed significantly increased levels of glucose (by 22%, *p* = 0.002), CRP (by 61%, *p* = 0.008), and LDH (by 47%, *p* = 0.001) in the plasma of STEMI patients with MACE compared to STEMI patients without MACE (Table 1). 

It is known that hyperglycemia is a risk factor for CVD in SA (*n* = 7/23) and UA (*n* = 10/21) patients, suggesting an aggravated biochemical plasma profile compared to normoglycemic patients. We found that levels of ApoA-I (by 30%, *p* = 0.028) and the enzymatic activity of PON1 (by 58%, *p* = 0.002) were significantly decreased in the plasma of SA-HG patients compared to SA-normoglycemic (SA-NG) patients. We also found that PON1 activity was decreased (by 58%, *p* = 0.042) while MPO protein levels were increased (by 55%, *p* = 0.044) in the plasma of UA-HG patients compared to UA-NG patients (Table S1, Supplementary Material).

Interestingly, we found biochemical parameters that could differentiate between SA and UA hyperglycemic patients, with significantly increased apolipoprotein A-I (ApoA-I) (by 47%, *p* = 0.026) levels and significantly decreased paraoxonase 1 (PON1) protein (by 38%, *p* = 0.008) levels in the plasma of UA-HG patients compared to SA-HG patients (by 47%, *p* = 0.026 for ApoA-I and by 61%, *p* = 0.008 for PON1 protein), while ApoE levels were higher between the same groups (by 67%, *p* = 0.036) (Table S1).

### 2.2. Distribution of ncRNAs in the Plasma of SA, UA, and STEMI Patients

**Figure 1:**
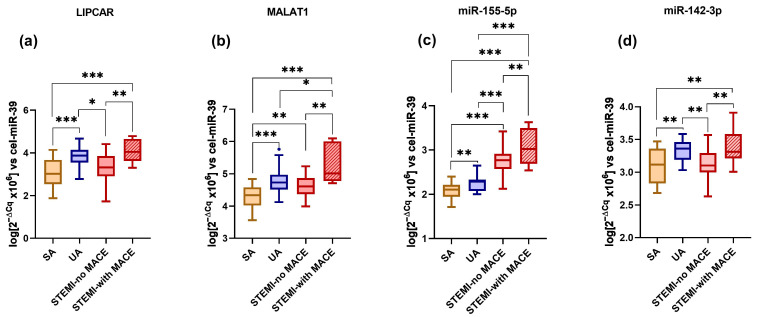
Levels of ncRNAs in the plasma of SA and UA patients with NG or HG and STEMI patients with or without MACE: LIPCAR (**a**), MALAT1 (**b**), miR-155-5p (**c**), miR-142-3p (**d**). Data are illustrated as boxplots with Tukey whiskers and median line. * *p* < 0.05; ** *p* < 0.01; *** *p* < 0.001.

**Figure 2:**
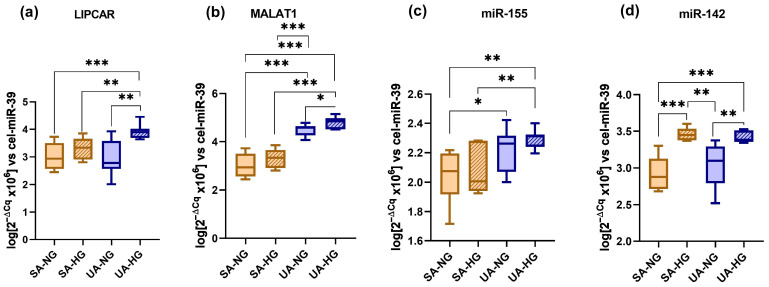
Levels of ncRNAs in the plasma of hyperglycemic (HG) SA and UA patients compared to normoglycemic (NG) patients: LIPCAR (**a**), MALAT1 (**b**), miR-155-5p (**c**), miR-142-3p (**d**). Data are illustrated as boxplots with Tukey whiskers and median line. * *p* < 0.05; ** *p* < 0.01; *** *p* < 0.001.

The distribution of ncRNAs in the plasma of SA, UA, and STEMI patients is presented in Figure 1. LIPCAR levels were significantly higher in the plasma of vulnerable CAD patients: UA (by 24%, *p* = 6.07 × 10^−4^) and STEMI-with MACE (by 32%, *p* = 3.46 × 10^−4^) compared to SA patients. Levels of LIPCAR were increased in the plasma of STEMI-with MACE patients compared to STEMI-no MACE patients (by 22%, *p* = 0.0025) (Figure 1a). Interestingly, LIPCAR levels were significantly increased in the plasma of UA-HG patients compared to SA-HG patients (by 18%, *p* = 0.0061) (Figure 2a).

MALAT1 levels were significantly increased in the plasma of UA (by 11%, *p* = 3.11 × 10^−4^), STEMI-no MACE (by 6%, *p* = 0.0079), and STEMI-with MACE (by 21%, *p* = 2.46 × 10^−5^) patients compared to the SA group, and they were also increased in STEMI-with MACE patients compared to both UA (by 9%, *p* = 0.04) and STEMI-no MACE (by 13%, *p* = 0.0013) patients (Figure 1b). MALAT1 levels in the plasma of UA-NG patients were increased compared to SA-NG (by 50%, *p* = 4.35 × 10^−7^) and SA-HG (by 36%, *p* = 3.21 × 10^−5^) patients. Plasma levels of MALAT1 in UA-HG patients were significantly increased compared to SA-NG (by 60%, *p* = 1.26 × 10^−7^) and SA-HG (by 46%, *p* = 9.81 × 10^−6^) patients (Figure 2b). 

Levels of miR-155-5p were significantly increased in the plasma of UA (by 7%, *p* = 0.004), STEMI-no MACE (by 32%, *p* = 1.24 × 10^−12^), and STEMI-with MACE (by 46%, *p* = 7.53 × 10^−11^) patients compared to SA patients. Additionally, miR-155-5p levels were increased in the plasma of STEMI-no MACE (by 22%, *p* = 3.90 × 10^−9^) and STEMI-with MACE (by 35%, *p* = 3.09 × 10^−9^) patients compared to UA patients, as well as in STEMI-with MACE patients vs. STEMI-no MACE patients (by 11%, *p* = 0.0065) (Figure 1c). Higher levels were detected in the plasma of UA-NG patients vs. SA-NG patients (by 7%, *p* = 0.047), as well as in the plasma of UA-HG patients compared to SA-HG (by 10%, *p* = 0.007) and SA-NG (by 12%, *p* = 0.0019) patients (Figure 2c). Additionally, miR-142-3p levels were increased in the plasma of SA-HG patients (by 13%, *p* = 0.009) compared to UA-NG patients, while UA-HG patients had higher levels than SA-NG patients (by 16%, *p* = 3.15 × 10^−4^).

Levels of miR-142-3p were significantly increased in UA (by 8%, *p* = 0.0036) and STEMI-with MACE patients (by 9%, *p* = 0.008) compared to SA patients, and also in the STEMI-with MACE group compared to the STEMI-no MACE group (by 7%, *p* = 0.0055), but they were decreased in the STEMI-no MACE group compared to UA patients (by 6%, *p* = 0.0036) (Figure 1d). Interestingly, miR-142-3p levels were significantly increased in HG patients compared to NG patients with SA (by 17%, *p* = 4.86 × 10^−4^) and UA (by 12%, *p* = 0.0081) (Figure 2d).

In conclusion, we found statistically significant differences in LIPCAR, MALAT1, and miR-142-3p levels between NG and HG patients in the UA group and only in miR-142-3p levels in SA patients (Figure 2). 

### 2.3. Correlations between lncRNAs, miRNAs, and Main Biochemical Parameters in Plasma of CAD Patients

**Figure 3:**
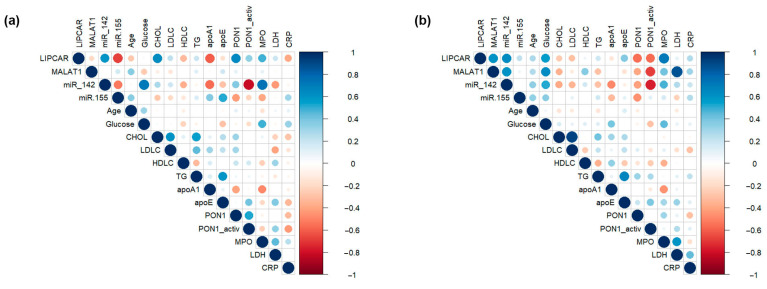
Correlation plots for stable (SA) (**a**) and unstable angina (UA) (**b**) patients. Bivariate Spearman’s nonparametric correlations between plasma lncRNA (LIPCAR, MALAT1) and miRNA (miR-142-3p, miR-155-5p) levels with age, biochemical parameters, enzymatic activity, and inflammatory stress-related parameters. Data were illustrated as correlation plot graphs using R-studio software (version 2022.12.0+353).

Correlations between plasma levels of selected lncRNAs, miRNAs, and main plasma parameters in CAD patients were estimated separately for the SA and UA groups using Pearson’s analysis. Significant correlations were observed for plasma lncRNA LIPCAR levels with lncRNA MALAT1 (R = 0.604, *p* = 0.029) and miR-142-3p (R = 0.638, *p* = 0.026) levels in UA patients, and with miR-142-3p (R = 0.596, *p* = 0.019) and miR-155-5p (R = −0.670, *p* = 0.009) levels in SA patients. Plasma lncRNA MALAT1 levels were significantly correlated with plasma miR-142-3p (R = 0.608, *p* = 0.042) levels only in UA patients (Figure 3 and Table S2, Supplementary Material).

When analyzing the associations of measured lncRNA levels with lipid metabolism-associated parameters, we observed significant and positive correlations between LIPCAR levels and total cholesterol (R = 0.633, *p* = 0.008) or ApoA-I (R = −0.602, *p* = 0.014) levels in the SA group. No significant correlations were found between plasma levels of MALAT1 and lipid metabolism-related parameters in SA or UA patients. In the plasma of SA patients, significant correlations were observed between miR-142-3p and ApoA-I levels (R = −0.569, *p* = 0.022), while miR-155-5p levels were positively correlated with ApoE levels (R = 0.516, *p* = 0.034) (Figure 3 and Table S3, Supplementary Material).

We also analyzed the correlations between lncRNA and miRNA levels with parameters associated with oxidative stress. Plasma LIPCAR levels were positively correlated with PON1 protein levels in SA patients (R = 0.645, *p* = 0.009), while a strong positive correlation was observed with MPO protein levels (R = 0.715, *p* = 6.01 × 10^−3^) in UA patients. Plasma MALAT1 levels showed a negative correlation with PON1 activity (R = −0.686, *p* = 0.016) in UA patients. Similarly, miR-142-3p levels were negatively correlated with PON1 activity in the plasma of both SA (R = −0.812, *p* = 0.014) and UA (R = −0.765, *p* = 0.031) patients (Figure 3 and Table S4, Supplementary Material).

We further analyzed the potential associations of lncRNA and miRNA levels with cardiac parameters, and we observed that MALAT1 levels had a strong positive correlation with LDH levels (R = 0.861, *p* = 0.010) only in UA patients (Figure 3 and Table S4, Supplementary Material). No significant correlations were observed between plasma levels of lncRNAs and miRNAs with an inflammatory stress-related parameter (CRP) either in SA or UA patients (Figure 3 and Table S4, Supplementary Material).

**Figure 4:**
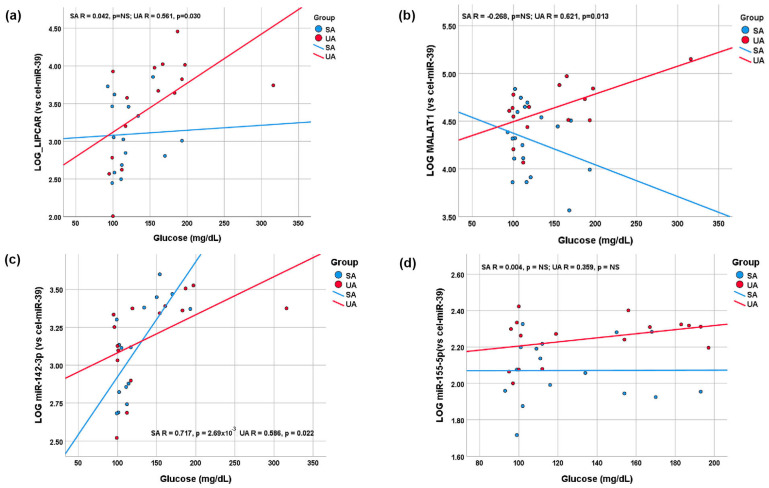
Scatter plots and linear regression curves. The associations between plasma levels of glucose and ncRNAs: LIPCAR (**a**), MALAT1 (**b**), miR-155-5p (**c**), and miR-142-3p (**d**) in stable (SA, blue dots and lines) and unstable angina (UA, red dots and lines) patients.

The most interesting associations were observed between the analyzed lncRNAs and miRNAs with glucose levels in the plasma of SA and UA patients, illustrated as scatter plots in Figure 4 and detailed in Table S3. LIPCAR and MALAT1 levels were positively correlated with glucose levels only in UA patients (R = 0.561, *p* = 0.030, and R = 0.621, *p* = 0.013, respectively) (Figure 4a,b and Table S3, Supplementary Material). Plasma miR-142-3p levels displayed strong correlations with glucose levels both in SA (R = 0.717, *p* = 2.68 × 10^−3^) and UA (R = 0.586, *p* = 0.022) patients. In contrast, no significant correlations were found between plasma miR-155-5p and glucose levels (Figure 4c,d and Table S3, Supplementary Material).

### 2.4. Correlations between lncRNAs, miRNAs, and Main Parameters in Plasma of STEMI Patients

**Figure 5:**
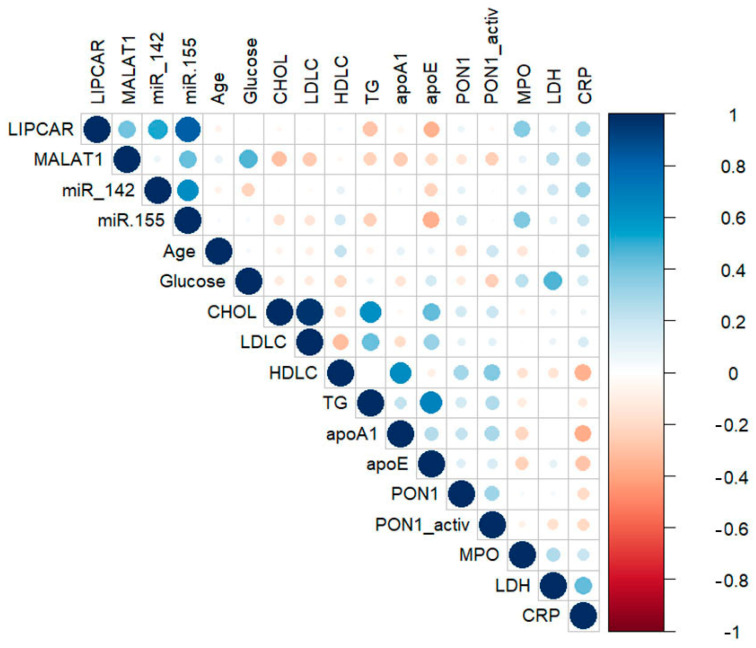
Correlation plot for ST-segment elevation myocardial infarction (STEMI) patients. Bivariate Spearman’s nonparametric correlations between serum lncRNA (LIPCAR, MALAT1) and miRNA (miR-142-3p, miR-155-5p) levels with age, biochemical parameters, enzymatic activity, and inflammatory stress-related parameter. Data were illustrated as a correlation plot graph using R-studio software (version 2022.12.0+353).

Similar correlations between lncRNAs and miRNAs with main plasma parameters were investigated in STEMI patients (Figure 5, and detailed in Table S5, Supplementary Material). We observed that plasma LIPCAR levels were positively correlated with MALAT1 (R = 0.405, *p* = 0.014), miR-142-3p (R = 0.528, *p* = 6.58 × 10^−4^), and miR-155-5p (R = 0.826, *p* = 1.70 × 10^−10^) levels in STEMI patients. Significant positive correlations were also observed between plasma miR-155-5p levels with MALAT1 (R = 0.424, *p* = 0.010) and miR-142-3p (R = 0.628, *p* = 2.16 × 10^−7^) levels (Figure 5 and Table S5, Supplementary Material).

We analyzed the correlations between lncRNA and miRNA levels with lipid metabolism-associated parameters in the plasma of STEMI patients, and we observed moderate significant correlations between LIPCAR and ApoE (R = −0.356, *p* = 0.028), MALAT1 and ApoA-I (R = −0.384, *p* = 0.025), and miR-155-5p and ApoE (R = −0.362, *p* = 6.15 × 10^−3^) levels. Only MALAT1 levels correlated with glucose levels (R = 0.468, *p* = 8.97 × 10^−3^) in the plasma of STEMI patients (Figure 5 and Table S6, Supplementary Material).

Correlations between parameters associated with oxidative stress in the plasma of STEMI patients were determined, with LIPCAR and miR-155-5p levels associated with MPO protein levels (R = 0.373, *p* = 0.025, and R = 0.380, *p* = 4.63 × 10^−3^, respectively). We further analyzed the associations with cardiac parameters in the plasma of STEMI patients, and we observed that only miR-142-3p levels showed a significant correlation with CRP levels (R = 0.316, *p* = 0.024) (Figure 5 and Table S7, Supplementary Material).

### 2.5. Plasma lncRNA and miRNA Levels as Predictors for Vulnerable CAD Patients: The Impact of Hyperglycemia

**Figure 6:**
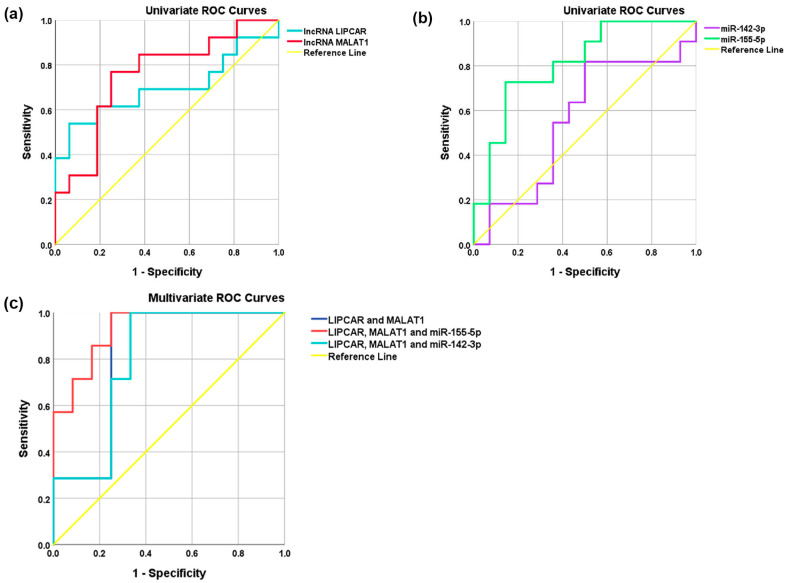
Receiver operator characteristic (ROC) curves. The discriminating potential of univariate and multivariate models for diagnosis of acute coronary syndrome—unstable angina (UA) versus stable angina (SA)—in patients using plasma levels of lncRNA LIPCAR, lncRNA MALAT1, miR-155-5p, or miR-142-3p (**a**,**b**), and multivariate models (**c**) using plasma levels of two lncRNAs or two lncRNAs plus miR-155-5p or miR-142-3p.

We estimated the prediction power of the analyzed ncRNAs for the prediction of vulnerability evolution in ACS patients using receiver operating characteristic (ROC) analysis, adjusted for age and gender. The reference was the SA group and the risk group was the UA group. Except for miR-142-3p, each of analyzed ncRNAs could be used as individual predictors for vulnerable CAD (UA diagnosis) in ACS patients in univariate ROC analysis, whereas miR-155-5p levels were shown to be the strongest independent predictor (highest AUC = 0.812, *p* = 3.55 × 10^−3^), followed by MALAT1 (AUC = 0.773, *p* = 3.27 × 10^−3^) and LIPCAR (AUC = 0.737, *p* = 0.022) levels (Table S8 and Figure 6).

The multivariate ROC analysis indicated that the combination of the two lncRNAs, LIPCAR and MALAT1, could strongly predict vulnerable CAD (multivariate model 1, AUC = 0.870, *p* = 7.34 × 10^−4^) (Table S8 and Figure 6). The addition of miR-155-5p levels to the multivariate model 1 significantly improved the prediction of vulnerable CAD (UA diagnosis) (multivariate model 2, AUC = 0.938, *p* = 4.08 × 10^−4^), while the addition of miR-142-3p levels failed to improve the model but the prediction still remained statistically significant (multivariate model 3, AUC = 0.836, *p* = 5.92 × 10^−3^) (Table S8 and Figure 6). According to these data, the minimal model for discriminating vulnerable CAD was based on plasma LIPCAR, MALAT1, and miR-155-5p levels.

**Figure 7:**
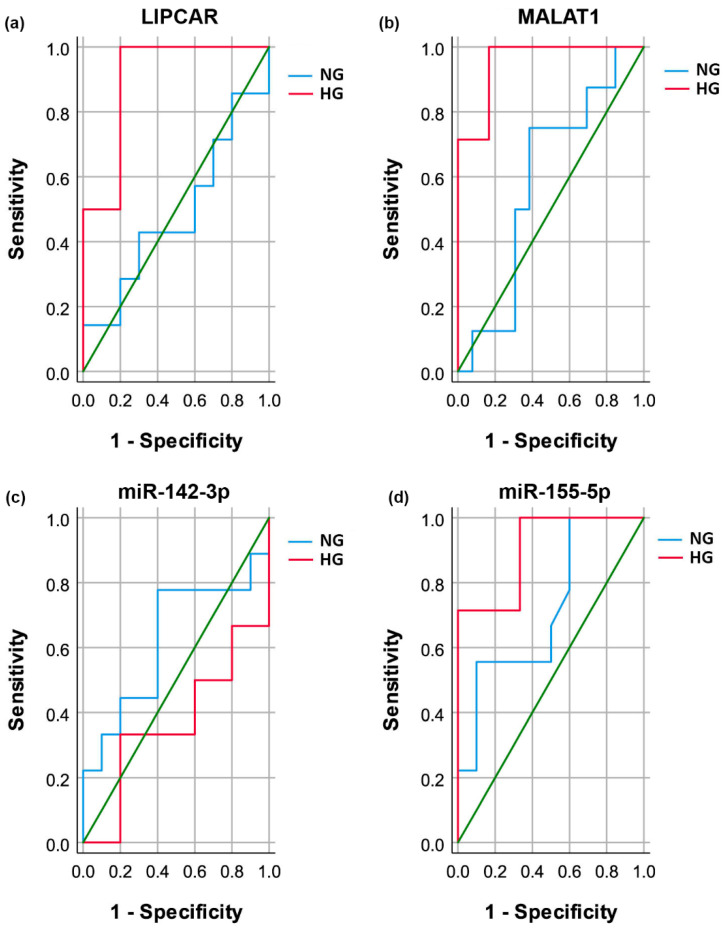
Receiver operator characteristic (ROC) curves. Univariate analysis for the discriminating potential of vulnerable coronary artery disease (CAD)—unstable angina (UA) versus stable angina (SA)—using plasma levels of LIPCAR (**a**), MALAT1 (**b**), miR-142-3p (**c**), and miR-155-5p (**d**) in hyperglycemic (HG) and normoglycemic (NG) CAD patients.

Using the same statistical approach of ROC analysis, we evaluated the discriminating potential of the selected lncRNA and miRNA levels for vulnerable CAD diagnosis (UA-risk group versus SA-reference group) in the two subgroups of CAD patients defined by the presence of hyperglycemia (Figure 7a–d and Table 2). We obtained significant and powerful predictions of vulnerable CAD, mostly in HG patients, with the highest AUC achieved using MALAT1 levels (AUC = 0.952, *p* = 6.66 × 10^−15^), followed by miR-155-5p (AUC = 0.905, *p* = 2.32 × 10^−6^) and LIPCAR (AUC = 0.900, *p* = 7079 × 10^−5^) levels. In contrast, miR-142-3p levels failed to discriminate vulnerable CAD in the HG group as well as in NG patients (Figure 7a–d and Table 2). Of note, miR-155-5p levels had a good AUC value in the ROC analysis for vulnerable CAD in NG patients but failed to reach statistical significance (AUC = 0.717, *p* = 0.121).

Further, we analyzed independent-group AUC differences in the ROC analysis between the NG and HG groups of CAD patients. We found that the discriminating potential for vulnerable CAD using plasma LIPCAR and MALAT1 levels was significantly higher in HG patients than in the NG group (z value = −2.272, *p* = 0.023, and z = −2.588, *p* = 0.010, respectively) (Table 3).

### 2.6. Plasma lncRNA and miRNA Levels as Predictors for MACE Occurrence in STEMI Patients

**Figure 8:**
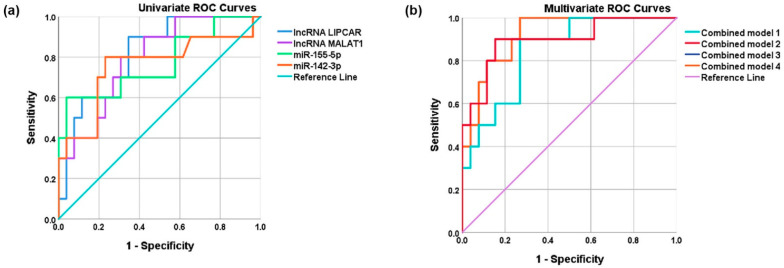
Receiver operator characteristic (ROC) curve. The predictive potential of univariate (**a**) and multivariate (**b**) models for major adverse cardiovascular events (MACEs) during follow-up in ST-elevation myocardial infarction (STEMI) patients using plasma levels of lncRNA LIPCAR, lncRNA MALAT1, miR-155-5p, or miR-142-3p (**a**) and combination models (**b**) using plasma levels of two lncRNAs (model 1) plus miR-155-5p (model 2), miR-142-3p (model 3), or the two miRNAs (model 4).

When analyzing the univariate ROC prediction for subsequent MACE in STEMI patients, we found that the selected lncRNAs and miRNAs had significant individual prognostic value. Accordingly, the highest AUC for MACE occurrence in STEMI patients was calculated for LIPCAR levels (AUC = 0.815, *p* = 0.004), followed by MALAT1 (AUC = 0.792, *p* = 0.007), miR-142-3p (AUC = 0.815, *p* = 0.004), and miR-155-5p (AUC = 0.815, *p* = 0.004) levels (Figure 8a and Table 4).

We then combined these ncRNAs in a multivariate ROC analysis and we proved that the combination of plasma levels of LIPCAR and MALAT1 could significantly predict future MACE in STEMI patients (AUC = 0.842, *p* = 0.002) (multivariate model 1 in Figure 8b and Table 4). The individual addition of miR-155-5p (multivariate model 2) and miR-142-3p levels (multivariate model 3) to model 1 significantly improved the prediction of new MACE (AUC = 0.896, *p* = 2.75 × 10^−4^, and AUC = 0.919, *p* = 1.18 × 10^−4^, respectively). Of note, the model with miR-142-5p levels had a higher AUC than that with miR-155-5p levels (Figure 8b and Table 4). The multivariate model using all analyzed ncRNAs did not further improve the prediction. Similar to the vulnerable CAD discrimination model, the minimal model predicting subsequent MACE in STEMI patients was based on LIPCAR and MALAT1 levels, with an improvement when using miR-142-3p levels.

## 3. Discussion

Understanding the underlying molecular mechanisms involved in CVD is key to the successful treatment of patients. In the present study, we set out to investigate the potential of circulating lncRNAs LIPCAR and MALAT1 to discriminate ACS patients at risk. Furthermore, we evaluated the additional contribution of miR-155-5p and miR-142-3p to the prognostic properties of the lncRNAs of interest.

In this study, we report that in SA and UA patients: (i) LIPCAR, MALAT1, miR-142-3p, and miR-155-5p levels are significantly elevated in the plasma of vulnerable CAD (UA) compared to SA patients; (ii) LIPCAR, MALAT1, and miR-142-3p levels are significantly increased in the plasma of UA-HG patients compared to NG ones, while miR-142-3p presents at higher levels in the plasma of SA-HG patients vs. SA-NG patients; (iii) LIPCAR, MALAT1, and miR-142-3p levels are positively correlated with glucose levels and oxidative stress parameters in UA patient plasma; (iv) LIPCAR, MALAT1, and miR-155-5p levels could be used as individual predictors for vulnerable CAD (UA) in univariate ROC analysis only for HG patients; (v) the minimal multivariate ROC model discriminating between vulnerable and stable CAD is based on plasma LIPCAR and MALAT1 levels, with minimal improvement by miR-155-5p addition.

The data obtained in STEMI patients show that: (i) plasma levels of LIPCAR, MALAT1, miR-155-5p, and miR-142-3p are significantly higher in STEMI patients with MACE than in those without MACE; (ii) MALAT1 levels correlate with glucose and LDH levels, while LIPCAR levels correlate with oxidative stress parameters; (iii) LIPCAR, MALAT1, miR-142-3p, and miR-155-5p levels have significant individual prognostic value in univariate ROC analysis for subsequent MACE in STEMI patients; (iv) the minimal multivariate ROC model predicting subsequent MACE in STEMI patients is based on LIPCAR and MALAT1 levels, with some improvement by adding miR-142-3p levels. The novelty of our study is that the combined use of two lncRNAs, LIPCAR and MALAT1, measured in the plasma of ACS patients, may discriminate vulnerable CAD from SA, correlate with hyperglycemia in UA patients, and predict unfavorable evolution (MACE) of STEMI patients.

The most interesting and new data obtained in the present study are about LIPCAR, since it is still a relatively newly discovered mitochondrial lncRNA [12] with few published data proving its functional role. We show here that LIPCAR levels are increased in vulnerable CAD patients compared to SA patients and are strongly associated with hyperglycemia in UA patients, being also predictive for MACE occurrence in STEMI patients. In good agreement with our data, other studies suggest that LIPCAR may have a role in predicting heart failure and cardiac remodeling [13] and in diagnosing UA [25]. Our data confirm the results of other studies performed in patients with well-controlled type 2 diabetes, showing that serum LIPCAR levels are associated with left ventricle diastolic function and remodeling [26]. Our results are in good agreement with the data of Li et al., who found that elevated LIPCAR levels may predict the severity and progression of CAD, being an independent predictor for MACE in STEMI patients [27]. The novelty of our study is that increased LIPCAR levels are associated with hyperglycemia in the plasma of UA patients.

However, little is known about the pathways in which the studied lncRNAs are involved. Published in vitro data suggested that LIPCAR is upregulated in exosomes derived from THP-1 monocytes incubated with oxidized LDL and that these exosomes could promote human umbilical endothelial cell (HUVEC) and smooth muscle cell (SMC) proliferation and, consequently, the progression of atherosclerosis [15]. Another published study showed that LIPCAR overexpression may promote vascular SMC proliferation, migration, and phenotypic switch [16]. We show here, for the first time, a strong statistical association between LIPCAR levels and hyperglycemia in UA patients and the strong potential of the LIPCAR-MALAT1 model for the prediction of unfavorable post-AMI evolution.

The other lncRNA analyzed in our study is MALAT1. We show that plasma levels of MALAT1 are associated with hyperglycemia in vulnerable CAD patients. We demonstrate that MALAT1 levels are independent predictors for subsequent MACE in STEMI patients, both in univariate and multivariate ROC models (combined with LIPCAR). In good agreement with our data, a recent study showed that increased MALAT1 levels in peripheral blood mononuclear cells (PBMCs) are related to elevated MACE incidence [28]. Vausort et al. demonstrated that MALAT1 levels in PBMCs are increased in AMI patients compared to healthy subjects and may help in the prediction of left ventricle dysfunction [29]. Our data are complementary to the results of Lv et al., who showed that serum MALAT1 levels are predictive for subsequent MACE in CAD patients without AMI [30]. The novelty of our study is that MALAT1 levels can discriminate vulnerable CAD in HG patients and can predict MACE occurrence in STEMI patients using easier to perform lncRNA measurements in plasma and not PBMCs assessment.

The main mechanisms for post-transcriptional regulation by MALAT1 involve alternative splicing, but MALAT1 can also act as a competitive endogenous RNA (ceRNA) [31]. Interestingly, mRNAs can communicate with lncRNAs through miRNA response elements (MREs). MALAT1 uses the ceRNA regulatory system through negative regulation between MALAT1 and miRNAs [32]. Accordingly, it was demonstrated that MALAT1 physically interacts with some miRNAs, like the two miRNAs selected and analyzed in the present study, miR-155-5p and miR-142-3p. Wang et al. reported that MALAT1 can promote cell proliferation and migration in hypoxic cardiac stem cells, acting mechanistically by promoting MEF2A expression via sponging miR-155 [33]. Also, Chen et al. showed recently that MALAT1 can regulate intermittent hypoxia-induced injury of HUVECs via targeting miR-142-3p by direct interaction, as proven by luciferase-reporter assays [34]. In good agreement with these data, we found an expected positive correlation between MALAT1 and miR-142-3p levels in UA patients but not in SA patients. Furthermore, we found several statistically significant correlations between MALAT1 and LIPCAR levels, some biochemical parameters related to oxidative stress, such as PON1 activity and MPO protein levels, and LDH levels only in the UA group, which further supports the diagnostic potential of lncRNAs for vulnerable CAD. Additionally, the two minimal ROC analysis statistical models using LIPCAR and MALAT1 levels to discriminate vulnerable CAD (UA vs. SA patients) and to predict MACE in STEMI patients could be moderately improved by adding miR-155-5p and miR-142-3p levels, respectively. Since we found a possible association between MALAT1 levels and miR-155-5p or miR-142-3p levels in plasma, we cannot exclude an interaction between them in cardiac or vascular cells that may regulate their plasma levels. Based on these data, we hypothesize that the association of MALAT1 with selected miRNAs may happen only in acute cardiac events.

One possible limitation of our study is the relatively small number of CAD and STEMI patients analyzed. Although the number of recruited CAD and STEMI patients from the previous studies was rather small, the employed statistical analyses and ROC models, as well as the distribution of lncRNAs and miRNAs levels, showed statistically significant results. Another potential drawback is that despite the well-designed measurement techniques used, there are no standardized methods to quantify the absolute levels of blood cell-free circulating (plasma compartment) lncRNAs and miRNAs, making the comparison between data obtained in different laboratories difficult.

Taking all these data together, we propose LIPCAR and MALAT1 as potential prognostic markers of vulnerability in CAD patients and as robust predictors for unfavorable evolution (MACE) of STEMI patients. These lncRNAs can be easily quantified in plasma using the fast and reliable TaqMan-based PCR method, which is available in most clinical hospitals. However, a deeper understanding of lncRNA functions and their interaction networks is needed. This would lead to the development of lncRNAs as promising therapeutic targets and as validated diagnostic or prognostic biomarkers for vulnerable CAD.

## 4. Materials and Methods

### 4.1. Study Design: SA, UA, and STEMI Patients

For the present study, we used plasma samples available in our biobank from previous studies [22,35]. A total of 94 patients were enrolled as follows: 23 SA patients (7 females and 16 males, aged between 44 and 79 years old), 21 UA patients (14 females and 7 males, aged between 42 and 70 years old), and 50 STEMI patients (all males, aged between 33 and 68 years old). SA and UA patients were enrolled at the Cardiology Clinic, Elias Emergency University Hospital, Bucharest, while STEMI patients were from the Floreasca Emergency Hospital, Bucharest, Romania.

Inclusion of patients in the SA, UA, or STEMI groups was performed according to the clinical practice guidelines of the European Society of Cardiology (ESC) [1,36] by clinical assessment, cardiac biomarkers levels, electrocardiography, and echocardiography. Patients presenting previous myocardial infarction, cardiac surgery, active malignancy, auto-immune diseases, severe hepatic/respiratory/renal failure, recent surgery, or trauma were excluded from the study.

STEMI patients were monitored for the occurrence of post-STEMI MACE. We defined MACE according to the guidelines described by the ESC [36] as recurrent ischemia/reinfarction requiring hospital admission and repeated percutaneous coronary intervention (PCI); heart failure requiring hospital readmission; or death from cardiovascular causes. STEMI patients were divided in 2 groups: “no MACE” (*n* = 38) and “with MACE” (*n* = 12).

Fasting blood samples were collected in EDTA-treated tubes upon admission to the hospital for SA and UA patients, while for STEMI patients, blood samples were collected upon discharge from the hospital, based on our previous study [22]. The highest levels of the measured parameters were determined at this time, compared to those at hospital admission or at the 6-month follow-up. None of the patients received heparin or fractioned heparin at the time of blood collection. Plasma was obtained by centrifugation at 2000× *g* for 10′ at 4 °C, aliquoted, and stored at −80 °C until further processing.

The study was carried out in adherence to the principles of the Declaration of Helsinki (Code of Ethics of the World Medical Association, last updated at the 64th WMA General Assembly, Fortaleza, Brazil, October 2013) for experiments involving humans. All of the participants in the study gave their written informed consent by signing the respective paperwork and keeping their anonymity and privacy rights. The Ethics Committee of the Institute of Cellular Biology and Pathology “Nicolae Simionescu” (#1382/17, September 2012; #03/08, October 2019) approved the previous studies.

### 4.2. Determination of Plasma Parameters of SA, UA, and STEMI Patients

The plasma parameters of the analyzed patients were measured using commercial kits. The TC, high density lipoprotein-cholesterol (HDL-C), low density lipoprotein-cholesterol (LDL-C), and triglycerides kits were from Dialab (Neudorf, Austria); the ELISA kits for ApoA-I and ApoE were from Mabtech (Nacka Strand, Sweden); the ELISA kits for PON1, myeloperoxidase (MPO), and CRP were from R&D Systems (Minneapolis, MN, USA); and the enzymatic activity kit for lactate dehydrogenase (LDH) was from Biovision (Waltham, MA, USA). All assays were performed according to the manufacturers’ instructions. PON1 enzymatic activity was measured using an adapted method in the plasma of the patients [2]. Glucose levels were measured by the hospital’s laboratory.

### 4.3. Analysis of ncRNAs in the Plasma of SA, UA, and STEMI Patients

Plasma ncRNAs were isolated using the miRNeasy Serum/Plasma kit (Qiagen, Hilden, Germany), following the manufacturer’s instructions. Twenty-five fmoles of synthetic cel-miR-39 (Life Technologies, Carlsbad, CA, USA) was added to each sample as a spike-in to correct for sample-to-sample variation, as previously described [22,37].

The isolated RNA was reverse-transcribed using the High-Capacity cDNA Reverse Transcription Kit (Applied Biosystems, Waltham, MA, USA). In order to reverse-transcribe both lncRNAs and miRNAs, we used random primers (supplied by the kit) as well as specific reverse-transcription primers for miR-142-3p and miR-155-5p on a Veriti 96-Well Fast Thermal Cycler (Applied Biosystems, USA).

Because of the known low abundance of lncRNAs in circulation, a preamplification step was needed before the quantification of LIPCAR and MALAT1 levels by qPCR. This was performed using SsoAdvanced PreAmp Supermix (Bio-Rad Laboratories, Hercules, CA, USA), according to the manufacturer’s instructions, on a Veriti 96-Well Fast Thermal Cycler (Applied Biosystems, USA).

Plasma expression levels of MALAT1 (00273907_s1), hsa-miR-142-3p (ID 000464), and hsa-miR-155-5p (ID 002623) were measured using the TaqMan assay method (Thermo Scientific, Waltham, MA, USA) according to the manufacturer’s instructions. This method included: a hold stage at 50 °C for 2 min; 95 °C for 10 min; 45 cycles of 95 °C for 10 s then 62 °C for lncRNAs or 60 °C for miRNAs for 1 min; followed by fluorescence readings using the ViiA7 Real-Time PCR System (Applied Biosystems, USA). For LIPCAR, we used custom primers and a custom-designed probe (See Table S9, Supplementary Material for detailed sequences). The expression level of each individual ncRNA was determined relative to that of exogenously added cel-miR-39 (ID 000200) and calculated using the 2^−ΔCq^ method [35].

### 4.4. Statistical Analysis

Statistical analysis and graphical representations of the study’s data were performed using SPSS software for Windows (IBM SPSS 26.0, IBM Ireland, Dublin, Ireland) and GraphPad Prism 9.0 (GraphPad Software Inc., San Diego, CA, USA). Correlation plots were designed using Statistical Software Package R 4.2.2 (particularly xlsx and corrplot packages) and R-studio for Windows (version 2022.12.0+353); scatter plots for correlations of ncRNAs with glucose levels were created using SPSS 26.0 Graph builder. To increase the power of the statistical analysis, we used log-transformed data for lncRNA and miRNA levels. The continuous distributed quantitative variables (age, biochemical, lncRNA, and miRNA data) were expressed as mean ± standard error of the mean (SEM) and analyzed using the independent Student’s *t*-test for comparisons between study groups. To evaluate the differences between categorical variables (gender, presence of hyperglycemia, hypertension, hyperlipidemia, diabetes, and obesity), we used Crosstabs distribution with chi-squared (χ^2^) analysis with Mantel–Haenszel common odds ratio (OR) estimates, performed using SPSS. Pearson’s parametric bivariate correlation analysis for plasma lncRNA and miRNA levels with age and biochemical parameters was performed using SPSS. To analyze the potential of plasma lncRNA and miRNA levels to discriminate vulnerable CAD patients, univariate and multivariate receiver operating characteristic (ROC) analysis (2-value) was performed, adjusted for age and gender (females as reference group), considering the SA group as the reference category and the UA group as the risk (vulnerable) category. Similarly, univariate and multivariate ROC analysis was used to estimate the prediction potential for subsequent MACE in STEMI patients, using plasma lncRNA and miRNA levels and considering the “no MACE” group as the reference category and the “with MACE” group as the risk (vulnerable) category, adjusting for age and gender (females as reference group). The threshold for statistical significance was considered as 5% (*p*-values lower than 0.05).

## Figures and Tables

**Table 1:** Clinical parameters in the plasma of stable angina (SA), unstable angina (UA), and ST-segment elevation myocardial infarction (STEMI) patients.

Parameters	SA (*n* = 23)	UA (*n* = 21)	STEMI	
			no MACE (*n* = 38)	with MACE (*n* = 12)
Total cholesterol (mg/dL)	168 ± 7.52	204 ± 11.17 *	181 ± 7.85	182 ± 11.38
HDL-C (mg/dL)	43 ± 1.74	45.11 ± 3.25	25.83 ± 1.09 ***^,###^	24.62 ± 1.39 ***^,###^
LDL-C (mg/dL)	95 ± 6.72	114 ± 8.13	127 ± 7.65 *	127 ± 10.81 *
Triglycerides (mg/dL)	166 ± 15.23	164 ± 17.73	140 ± 6.98	155 ± 17.13
ApoA-I (mg/dL)	151 ± 9.11	146 ± 8.10	98 ± 5.99 ***^,###^	92 ± 9.17 ***^,###^
ApoE (mg/dL)	2.16 ± 0.15	2.89 ± 0.28 *	2.32 ± 0.15 ^#^	2.31± 0.21
Glucose (mg/dL)	122 ± 6.23	135 ± 8.49	100 ± 3.05 **^,###^	122 ± 7.67 ^$$^
PON1 protein (μg/mL)	4.40 ± 0.39	3.65 ± 0.33	2.76 ± 0.12 ***^,##^	2.58 ± 0.20 **^,#^
PON1 activity (U/L)	446 ± 61.61	364 ± 61.47	380 ± 44.92	290 ± 68.53
MPO protein (μg/mL)	36.7 ± 2.84	42.7 ± 4.22	56.5 ± 6.16 *	67.1 ± 12.52 **^,#^
CRP (μg/mL)	17.0 ± 2.69	35.5 ± 6.99 *	22.6 ± 2.29 ^#^	36.6 ± 4.40 is **^,$$^
LDH (U/L)	1547 ± 94.16	1990 ± 213.6	4454 ± 259.08 ***^,###^	6589 ± 700.15 is ***^,###,$$^

Data are given as mean ± SEM. Variations between the parameters of “SA”, “UA”, “STEMI-no MACE”, and “STEMI-with MACE” groups were analyzed using independent Student’s *t*-tests and considered statistically significant when the *p*-value was below 0.05 (* vs. SA; # vs. UA), below 0.01 (** vs. SA; ## vs. UA; $$ vs. STEMI-no MACE), or below 0.001 (*** vs. SA; ### vs. UA). HDL-C, high-density lipoprotein cholesterol; LDL-C, low-density lipoprotein cholesterol; ApoA-I, apolipoprotein A-I; ApoE, apolipoprotein E; PON1, paraoxonase 1; MPO, myeloperoxidase; CRP, C-reactive protein; LDH, lactate dehydrogenase; SEM, standard error of the mean.

**Table 2:** Area under the receiver operator characteristic (ROC) curves of univariate analysis for the discriminating potential of acute coronary syndrome—unstable angina (UA) versus stable angina (SA)—using plasma levels of lncRNA LIPCAR, lncRNA MALAT1, miR-142-3p, and miR-155-5p in hyperglycemic (HG) and normoglycemic (NG) CAD patients.

Area Under the ROC Curve—UA vs. SA Analysis						
Test Result Variable(s)	Glycemia	Area	Std. Error ^a^	*p*-Value ^b^	Asymptotic 95% Confidence Interval	
					Lower Bound	Upper Bound
LIPCAR	Normoglycemic group	0.486	0.152	0.925	0.188	0.783
	Hyperglycemic group	0.900	0.101	7.79 × 10^−5^	0.702	1.098
MALAT1	Normoglycemic group	0.587	0.129	0.502	0.334	0.839
	Hyperglycemic group	0.952	0.058	6.66 × 10^−15^	0.839	1.066
miR-142-3p	Normoglycemic group	0.622	0.137	0.372	0.354	0.891
	Hyperglycemic group	0.367	0.176	0.448	0.022	0.711
miR-155-5p	Normoglycemic group	0.717	0.121	0.074	0.479	0.954
	Hyperglycemic group	0.905	0.086	2.32 × 10^−6^	0.737	1.073

^a^ Under the nonparametric assumption; ^b^ Null hypothesis: true area = 0.5.

**Table 3:** Comparative analysis of area under the receiver operator characteristic (ROC) curves between univariate analysis in hyperglycemic (HG) and normoglycemic (NG) patients for the discriminating potential of acute coronary syndrome—unstable angina (UA) versus stable angina (SA)—using plasma levels of lncRNA LIPCAR, lncRNA MALAT1, miR-142-3p, and miR-155-5p.

Independent-Group Area Difference Under the ROC Curve						
Test Result Variable(s)	Asymptotic		AUC Difference	Std. Error Difference ^b^	Asymptotic 95% Confidence Interval	
	z	*p*-Value ^a^			Lower Bound	Upper Bound
LIPCAR	−2.272	0.023	−0.414	0.182	−0.772	−0.057
MALAT1	−2.588	0.010	−0.366	0.141	−0.643	−0.089
miR-142-3p	1.147	0.251	0.256	0.223	−0.181	0.692
miR-155-5p	−1.268	0.205	−0.188	0.148	−0.479	0.103

^a^ Null hypothesis: true area difference = 0; ^b^ Under the nonparametric assumption.

**Table 4:** Receiver operator characteristic (ROC) analysis for predictive potentials of individual and combined models for major adverse cardiovascular events (MACEs) during follow-up in ST-elevation myocardial infarction (STEMI) patients, using plasma lncRNA LIPCAR, lncRNA MALAT1, miR-155-5p, and miR-142-3p levels.

Area Under the Curve					
Test Result Variable(s) *	Area	Standard Error ^a^	*p*-Value ^b^	Asymptotic 95% Confidence Interval	
				Lower Bound	Upper Bound
**Univariate models**					
LIPCAR	0.815	0.074	0.004	0.670	0.961
MALAT1	0.792	0.078	0.007	0.640	0.945
miR-155-5p	0.769	0.098	0.013	0.577	0.962
miR-142-3p	0.756	0.103	0.019	0.554	0.958
**Multivariate models**					
Multivariate model 1 (LIPCAR and MALAT1)	0.842	0.068	0.002	0.710	0.975
Multivariate model 2 (LIPCAR, MALAT1, and miR-155-5p)	0.896	0.064	2.75 × 10^−4^	0.771	1.000
Multivariate model 3 LIPCAR, MALAT1, and miR-142-3p)	0.919	0.045	1.18 × 10^−4^	0.832	1.000
Multivariate model 4 (lncRNAs LIPCAR and MALAT1, plus miR-142-3p and miR-155-5p)	0.919	0.045	1.18 × 10^−4^	0.832	1.000

* All adjusted for age and gender (male as risk). ^a^ Under the nonparametric assumption. ^b^ Null hypothesis: true area = 0.5.

## Data Availability

The datasets used and/or analyzed during the current study are available from the corresponding author on reasonable request.

## Supplementary Materials

The supporting information can be downloaded at: https://www.mdpi.com/article/10.3390/ijms241512076/s1.
