# Supplementary material for: Elevated Levels of Circulating lncRNAs LIPCAR and MALAT1 Predict an Unfavorable Outcome in Acute Coronary Syndrome Patients
Source: Int J Mol Sci. 2023 Jul 28;24(15):12076. doi: 10.3390/ijms241512076 (PMC10418585; doi:10.3390/ijms241512076)
Supplement: Supplementary file 1 [file ijms-24-12076-s001.zip › ijms-2477789-supplementary.pdf]

Elevated levels of circulating lncRNAs LIPCAR and MALAT1 predict an unfavorable outcome in acute coronary syndrome patients

Teodora Barbălată [1,#], Loredan S. Niculescu [1,#], Camelia S. Stancu [1], Florence Pinet [2] and Anca V. Sima [1,*]

[1] Lipidomics Department, Institute of Cellular Biology and Pathology "Nicolae Simionescu" of the Romanian Academy, 8, B.P. Hasdeu Street, 050568 Bucharest, Romania;

[2] INSERM, University of Lille, CHU Lille, Institut Pasteur de Lille, U1167 - RID-AGE - Facteurs de risque et déterminants moléculaires des maladies liées au vieillissement, F-59000 Lille, France

# These authors contributed equally.

* Corresponding author: anca.sima@icbp.ro

Table S1. Clinical parameters in the plasma of hyperglycemic (HG) stable angina (SA) and unstable angina (UA) compared to normoglycemic (NG) patients.

Parameters	SA-NG (n=16)	SA-HG (n=7)	UA-NG (n=11)	UA-HG (n=10)
**Total cholesterol (mg/dL)**	167 ± 8.2	171 ± 17.49	200 ± 15.89	208 ± 14.47*
**HDL-C (mg/dL)**	45 ± 1.94	37 ± 2.47	39 ± 2.65	51 ± 5.37
**LDL-C (mg/dL)**	95 ± 9.21	89 ± 9.45	112 ± 11.52	116 ± 10.73
**Triglycerides (mg/dL)**	164 ± 19.78	171 ± 22.15	184 ± 22.72	227 ± 54.24
**ApoA-I (mg/dL)**	154 ± 11.96	108 ± 9.85*	161 ± 15.37$^{\#}$	159 ± 13.71$^{\#}$
**ApoE (mg/dL)**	2.25 ± 0.20	1.96 ± 0.27	2.50 ± 0.30	3.27 ± 0.42$^{*, \#}$
**Glucose (mg/dL)**	105 ± 2.21	156 ± 9.07***	103 ± 2.41$^{\#\#\#}$	188 ± 15.05$^{***, \$\$\$}$
**PON1 protein (μg/mL)**	4.86 ± 0.59	4.46 ± 0.41	4.11 ± 0.48	2.76 ± 0.35$^{*. \#\#}$
**PON1 activity (U/L)**	557 ± 71.27	232 ± 34.5**	533 ± 110.03$^{\#}$	225 ± 65.04$^{**,\$}$
**MPO protein (μg/mL)**	31.9 ± 2.99	42.08 ± 3.62	34.67 ± 2.65	54.06 ± 10.19$^{*,\$}$
**CRP (μg/mL)**	15.43 ± 3.92	16.50 ± 3.39	33.99 ± 4.95$^{**, \#}$	38.96 ± 17.02
**LDH (U/L)**	1656 ± 151.5	1615 ± 139.29	1757 ± 177.85	2222 ± 383.01

Data are given as mean ± SEM. Variations between the parameters of "SA-NG", "SA-HG", "UA-NG" and "UA-HG" groups were analyzed by Independent Student T-test and considered statistically significant when the p value is below 0.05 (marked with * vs. SA-NG; # vs. SA-HG; $ vs. UA-NG), or below 0.01 (marked with ** vs. SA-NG; ## vs. SA-HG; $$ vs. UA-NG), or below 0.001 (marked with *** vs. SA-NG; ### vs. SA-HG; $$$ vs.UA-NG). HDL-C, high-density lipoprotein cholesterol; LDL-C, low-density lipoprotein cholesterol; apoA-I, apolipoprotein A-I; apoE, apolipoprotein E; PON1, paraoxonase 1; MPO, myeloperoxidase; CRP, C-reactive protein; LDH, lactate dehydrogenase; SEM, standard error of the mean.

Table S2. Bivariate Pearson's correlations between lncRNAs (LIPCAR and MALAT1) and miRNAs (miR-142-3p and miR-155-5p) levels in plasma of coronary artery disease (CAD) patients with stable (SA) or unstable angina (UA).

Parameter	Correlation	LIPCAR		MALAT1		miR-142-3p		miR-155-5p	
		SA	UA	SA	UA	SA	UA	SA	UA
**LIPCAR**	**R**	1	1	-0.201	0.604*	0.596*	0.638*	-0.670*	0.221
	**p value**	-	-	0.456	0.029	0.019	0.026	0.009	0.514
**MALAT1**	**R**	-0.201	0.604*	1	1	-0.071	0.608*	0.353	0.067
	**p value**	0.456	0.029	-	-	0.800	0.042	0.179	0.844
**miR-142-3p**	**R**	0.596*	0.638*	-0.071	0.608*	1	1	-0.529	-0.101
	**p value**	0.019	0.026	0.800	0.042	-	-	0.052	0.768
**miR-155-5p**	**R**	-0.670*	0.221	0.353	0.067	-0.529	-0.101	1	1
	**p value**	0.009	0.514	0.179	0.844	0.052	0.768	-	-

Table S3. Bivariate Pearson's correlations for lncRNAs (LIPCAR and MALAT1) and miRNAs (miR-142-3p and miR-155-5p) levels with age, glucose levels and lipid metabolism parameters in plasma of coronary artery disease (CAD) patients with stable (SA) or unstable angina (UA).

Parameter	Correlation	LIPCAR		MALAT1		miR-142-3p		miR-155-5p	
		SA	UA	SA	UA	SA	UA	SA	UA
Age (years)	R	-0.277	0.270	0.359	0.267	-0.076	0.340	0.354	0.314
	p value	0.281	0.331	0.120	0.336	0.771	0.216	0.219	0.149
Glucose (mg/dL)	R	0.042	0.561*	-0.268	0.621*	0.717**	0.586*	0.004	0.359
	p value	0.881	0.030	0.267	0.013	$2.69x10^{-3}$	0.022	0.960	0.172
Total cholesterol (mg/dL)	R	0.633*	-0.263	-0.101	-0.356	0.274	-0.386	-0.272	0.003
	p value	0.008	0.343	0.682	0.193	0.287	0.155	0.275	0.960
LDL-C (mg/dL)	R	0.479	-0.303	-0.136	-0.119	0.187	-0.342	-0.205	0.094
	p value	0.083	0.292	0.616	0.684	0.521	0.276	0.430	0.760
HDL-C (mg/dL)	R	-0.330	-0.174	-0.084	0.395	-0.361	0.171	-0.162	0.038
	p value	0.229	0.551	0.749	0.163	0.205	0.595	0.535	0.903
Triglycerides (mg/dL)	R	0.375	0.338	0.126	-0.321	0.050	-0.291	0.146	0.184
	p value	0.207	0.283	0.630	0.263	0.865	0.334	0.603	0.529
ApoA-I (mg/dL)	R	-0.602*	-0.279	-0.098	0.239	-0.569*	-0.486	0.379	-0.326
	p value	0.014	0.314	0.699	0.391	0.022	0.066	0.146	0.235
ApoE (mg/dL)	R	-0.174	0.472	0.243	-0.160	-0.299	-0.096	0.516*	0.105
	p value	0.518	0.103	0.316	0.586	0.261	0.755	0.034	0.710

Table S4. Bivariate Pearson's correlations for lncRNAs (LIPCAR and MALAT1) and miRNAs (miR-142-3p and miR-155-5p) levels with oxidative stress, cardiac (LDH) and inflammation (CRP) related parameters in plasma of coronary artery disease (CAD) patients with stable (SA) or unstable angina (UA).

**Parameter**	**Correlation**	**LIPCAR**		**MALAT1**		**miR-142-3p**		**miR-155-5p**	
		**SA**	**UA**	**SA**	**UA**	**SA**	**UA**	**SA**	**UA**
**PON1 protein (μg/mL)**	**R**	0.645*	-0.550	0.106	-0.394	0.370	-0.419	-0.423	-0.460
	**p value**	0.009	0.064	0.677	0.183	0.175	0.155	0.102	0.098
**PON1 activity (U/L)**	**R**	0.322	-0.563	0.038	-0.686*	-0.812*	-0.765*	-0.226	0.145
	**p value**	0.422	0.189	0.913	0.016	0.014	0.031	0.530	0.732
**MPO protein (ug/mL)**	**R**	0.488	0.715**	0.103	0.408	0.749**	0.583*	-0.393	0.160
	**p value**	0.068	$6.01x10^{-3}$	0.705	0.167	$3.18x10^{-3}$	0.040	0.164	0.638
**LDH (U/L)**	**R**	0.197	0.212	-0.162	0.861*	-0.433	0.269	-0.034	-0.240
	**p value**	0.639	0.686	0.634	0.010	0.284	0.560	0.590	0.568
**CRP (μg/mL)**	**R**	-0.389	0.278	0.044	0.375	-0.119	0.206	0.310	0.263
	**p value**	0.238	0.470	0.886	0.320	0.697	0.568	0.327	0.494

Table S5. Bivariate Pearson's correlations between lncRNAs (LIPCAR and MALAT1) and miRNAs (miR-142-3p and miR-155-5p) levels in plasma of patients with ST-elevation myocardial infarction (STEMI).

Parameter	Correlation	LIPCAR	MALAT1	miR-142-3p	miR-155-5p
**LIPCAR**	**R**	1	0.405*	0.528***	0.826***
	**p value**		0.014	6.58 $x10^{-4}$	1.70 $x10^{-10}$
**MALAT1**	**R**	0.405*	1	0.077	0.424*
	**p value**	0.014		0.656	0.010
**miR-142-3p**	**R**	0.528**	0.077	1	0.628***
	**p value**	6.58 $x10^{-4}$	0.656		2.16 $x10^{-7}$
**miR-155-5p**	**R**	0.826***	0.424*	0.628***	1
	**p value**	1.70 $x10^{-10}$	0.010	2.16 $x10^{-7}$	

Table S6. Bivariate Pearson's correlations for lncRNAs (LIPCAR and MALAT1) and miRNAs (miR-142-3p and miR-155-5p) levels with age, glucose levels and lipid metabolism parameters in plasma of patients with ST-elevation myocardial infarction (STEMI).

Parameter	Correlation	LIPCAR	MALAT1	miR-142-3p	miR-155-5p
**Age (years)**	**R**	-0.067	0.087	-0.165	0.032
	**p value**	0.692	0.613	0.251	0.814
**Glucose (mg/dL)**	**R**	0.022	0.468***	-0.230	0.039
	**p value**	0.898	8.97×10^{-3}	0.101	0.786
**Total cholesterol (mg/dL)**	**R**	-0.158	-0.308	-0.041	-0.170
	**p value**	0.357	0.076	0.784	0.219
**LDL-C (mg/dL)**	**R**	0.022	-0.264	-0.029	-0.149
	**p value**	0.901	0.132	0.834	0.281
**HDL-C (mg/dL)**	**R**	0.112	-0.243	0.097	0.170
	**p value**	0.547	0.204	0.483	0.218
**Triglycerides (mg/dL)**	**R**	-0.286	-0.232	-0.068	-0.242
	**p value**	0.091	0.186	0.644	0.078
**ApoA-I (mg/dL)**	**R**	-0.250	-0.384*	-0.148	-0.187
	**p value**	0.142	0.025	0.314	0.204
**ApoE (mg/dL)**	**R**	-0.356*	-0.205	-0.236	-0.362*
	**p value**	0.028	0.230	0.088	6.15×10^{-3}

Table S7. Bivariate Pearson's correlations for lncRNAs (LIPCAR and MALAT1) and miRNAs (miR-142-3p and miR-155-5p) levels with oxidative stress, cardiac (LDH) and inflammation (CRP) related parameters in plasma of patients with ST-elevation myocardial infarction (STEMI).

Parameter	Correlation	LIPCAR	MALAT1	miR-142-3p	miR-155-5p
**PON1 protein (μg/mL)**	**R**	0.079	-0.148	0.093	0.147
	**p value**	0.652	0.410	0.509	0.293
**PON1 activity (U/L)**	**R**	-0.046	-0.244	0.029	0.024
	**p value**	0.801	0.194	0.840	0.871
**MPO protein (ug/mL)**	**R**	0.373*	0.086	0.126	0.380**
	**p value**	0.025	0.627	0.365	4.63×10^{-3}
**LDH (U/L)**	**R**	0.071	0.246	0.181	0.105
	**p value**	0.677	0.154	0.194	0.456
**CRP (μg/mL)**	**R**	0.296	0.257	0.316*	0.202
	**p value**	0.094	0.156	0.024	0.155

Table S8. Receiver operator characteristic (ROC) analysis for the discriminating potentials of univariate and multivariate models for diagnosis of acute coronary syndrome - unstable angina (**UA**) versus stable angina (**SA**) in patients, using plasma lncRNAs LIPCAR, lncRNA MALAT1, miR-155-5p and miR-142-3p values.

Area Under the Curve					
**Test Result Variable(s)***	**Area**	**Standard Error** [a]	**p-value** [b]	**Asymptotic 95% Confidence Interval**	
				**Lower Bound**	**Upper Bound**
**Univariate models**					
LIPCAR	0.737	0.094	0.022	0.554	0.921
MALAT1	0.773	0.080	6.27×10^{-3}	0.617	0.930
miR-155-5p	0.812	0.087	3.55×10^{-3}	0.643	0.944
miR-142-3p	0.596	0.104	0.355	0.393	0.799
**Multivariate models**					
Multivariate model 1 (LIPCAR and MALAT1)	0.870	0.065	7.34×10^{-4}	0.742	0.999
Multivariate model 2 (LIPCAR, MALAT1 and miR-155-5p)	0.938	0.047	4.08×10^{-4}	0.846	1.000
Multivariate model 3 (LIPCAR, MALAT1 and miR-142-3p)	0.836	0.081	5.92×10^{-3}	0.677	0.994
* All adjusted for age and gender (male as risk). A. Under the nonparametric assumption. B. Null hypothesis: true area = 0.5.					

Table S9. LIPCAR custom primers and probe sequences used for LIPCAR quantification in the plasma by RT-qPCR.

Primer	Sequence
**Forward primer**	5'-TAATTGTCTGGGTCGCCTGG-3'
**Reverse primer**	5'-AGGTCAACGATCCCTCCCTT-3'
**Probe**	5-FAM-TCCGCCGTAGTCGGTGTACTCGTA-MGB-3’
